# Novel mutations in the *PLCZ1* gene associated with human low or failed fertilization

**DOI:** 10.1002/mgg3.1470

**Published:** 2020-08-24

**Authors:** Ping Yuan, Lingyan Zheng, Hao Liang, Qiyuan Lin, Songbang Ou, Yuqin Zhu, Luhua Lai, Qingxue Zhang, Zuyong He, Wenjun Wang

**Affiliations:** ^1^ IVF Center Department of Obstetrics and Gynecology Sun Yat‐sen Memorial Hospital Sun Yat‐sen University Guangzhou China; ^2^ Peking‐Tsinghua Center for Life Sciences Academy for Advanced Interdisciplinary Studies Peking University Beijing China; ^3^ Drug Clinical Trial Center Peking University Third Hospital Beijing China; ^4^ State Key Laboratory of Biocontrol School of Life Sciences Sun Yat‐sen University Guangzhou China; ^5^ Beijing National Laboratory for Molecular Sciences State Key Laboratory for Structural Chemistry of Unstable and Stable Species College of Chemistry and Molecular Engineering Peking University Beijing China

**Keywords:** fertilization failure, infertility, low fertilization, mutation, *PLCZ1*

## Abstract

**Background:**

Fertilization failure (FF) is a complex reproductive disorder characterized by the failure of pronuclei formation during fertilization. In addition to some cases caused by iatrogenic problems and known genetic factors, there are still many unexplained aspects of FF. Here, we aimed to assess the clinical and genetic characteristics of two families experiencing primary infertility with FF.

**Methods:**

We have characterized two families from China. All of the infertile couples presented with similar clinical phenotypes, that is, partial or total fertilization failure in repeated cycles. We performed Sanger sequencing of their *WEE2*, *TLE6*, and *PLCZ1* genes, and further bioinformatics and functional analyses were performed to identify the pathogenic elements of the variants.

**Results:**

We identified novel compound heterozygous mutations c.1259C>T (p.P420L) and c.1733T>C (p.M578T) in the *PLCZ1* gene in a male patient of family 1 with total fertilization failure, and another novel homozygous mutation c.1727T>C (p.L576P) in the same gene in a male patient of family 2 with partial fertilization failure. These three novel mutations were absent in the control cohort and in the databases. The amino acids were conserved at their positions among six different species. All mutant amino acids were located in key domains and were predicted to impair hydrolytic activity and lead to *PLCZ1* dysfunction. Further functional detection revealed that the three mutations could significantly impair the catalytic activity of *PLCZ1*.

**Conclusions:**

We identified three novel mutations in *PLCZ1* associated with partial and total fertilization failure and have provided new evidence about the genetic basis of FF.

## Graphical abstract

Fertilization failure (FF) is a complex reproductive disorder characterized by the failure of pronuclei formation during fertilization, which are still many unexplained aspects. We presented two infertility families with low fertilization or total fertilization failure in repeated cycles and identified three novel mutations in *PLCZ1* and provided further new evidence for the genetic basis of FF.

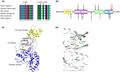

## 1 INTRODUCTION

The indicator of normal fertilization during in vitro fertilization (IVF) is the second polar body extrusion and the formation of male and female pronuclei (two‐pronuclear [2PN] zygote), and then, the embryos initiate early mitosis cell cycles (Anifandis, Messini, Dafopoulos, Sotiriou, & Messinis, 2014; Yeste, Jones, Amdani, Patel, & Coward, 2016). The key stages of fertilization are sperm‐oocyte fusion and the activation of the oocyte. Therefore, oocyte activation deficiency (OAD) is generally regarded as the principal cause of fertilization failure (Yeste et al., 2016). Although the intracytoplasmic sperm injection (ICSI) technique can improve the fertilization rate after IVF failure, there are still 1%–5% of ICSI cycles with total fertilization failure (TFF) (Yeste et al., 2016). However, using artificial oocyte activation (AOA) to overcome the above phenomena has resulted in partial success (Darwish & Magdi, 2015; Nikiforaki et al., 2016; Vanden Meerschaut, Nikiforaki, Heindryckx, & De Sutter, 2014).

Recently, some genetic defects associated with non‐teratozoospermia fertilization failure (FF) were characterized. There are three known genes responsible for FF, namely, phospholipase C zeta 1 (*PLCZ1*, MIM: 608075), WEE2 oocyte meiosis inhibiting kinase (*WEE2*, MIM: 614084) and TLE family member 6, the subcortical maternal complex member (*TLE6*, MIM: 612399) (Alazami et al., 2015; Escoffier et al., 2016; Sang et al., 2018). *PLCZ1*‐related FF can be attributed to male factors, but *WEE2*‐ and *TLE6*‐related FF are female factors. In the former case, chemical AOA can improve the fertilization rate, but it is not always effective for *WEE2*‐related FF (Dai et al., 2019; Torra‐Massana et al., 2019). *PLCZ1*‐related FF involves a different pathological mechanism, and Ca^2+^ oscillations are considered to be a key factor for PLCζ and AOA (Yeste et al., 2016). Furthermore, there are still many unexplained FF cases that need further investigation.

In this study, we described two families with clinical manifestations suggestive of FF. Three novel mutations in the *PLCZ1* gene were found. This study provides additional evidence for the role of *PLCZ1* in FF and advances our understanding of the clinical features of *PLCZ1* mutations.

## 2 METHODS

### 2.1 Patients and control populations

**Figure 1:**
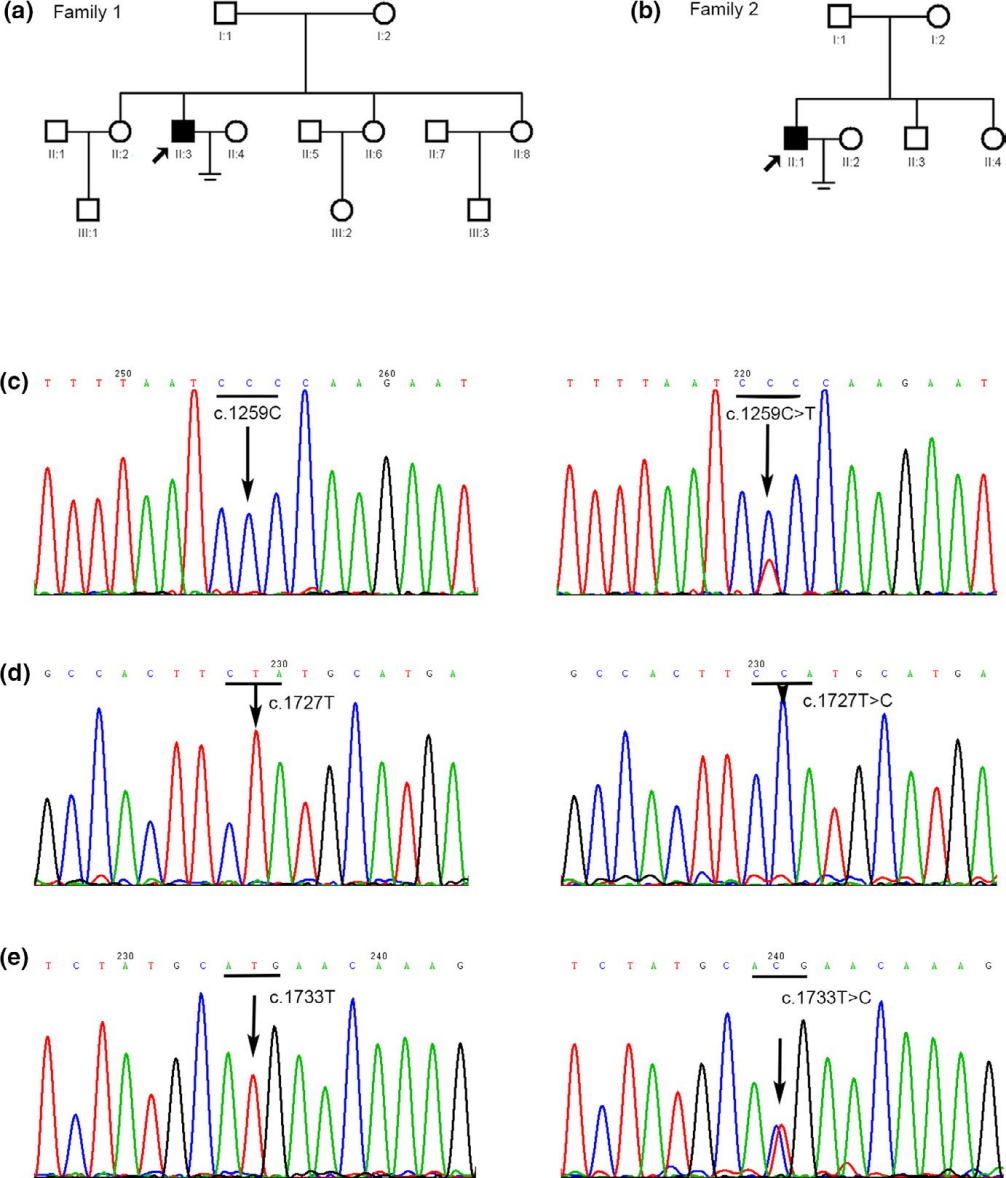
Partial sequencing results of novel mutations in FF patients for the *PLCZ1* gene. (a) Pedigree of the first family. Filled square indicates the FF patient (II:3). Arrow indicates the proband. Open squares or circles indicate normal family members. (b) Pedigree of the second family. Filled square indicates the FF patient (II:1). Arrow indicates the proband. Open squares or circles indicate normal family members. (c) The left arrow points to the wild‐type c.1259C in a control sample and the wild‐type codon is underlined; the right arrow points to the heterozygous c.1259C>T (p.P420L) mutation in the patient of family 1 (proband), and the mutated codon is underlined. (d) The left arrow points to the wild‐type c.1727T in a control sample, and the right arrow points to the homozygous c.1727T>C (p.L576P) mutation in the patient of family 2 (proband). (e) The left arrow points to the wild‐type c.1733T in a control sample, and the right arrow points to the heterozygous c.1733T>C (p.M578T) mutation in the patient of family 1 (proband), and the mutated codon is underlined

We evaluated two families with primary infertility with FF (Figure 1a,b) from the Sun Yat‐sen Memorial Hospital of Sun Yat‐sen University. Similarly, 200 healthy unrelated volunteers, who were fertile (father of at least one child) and had normal semen parameters, were recruited as controls. The study was approved by the Ethics Committee of Human Study at the Sun Yat‐sen Memorial Hospital of Sun Yat‐sen University, and the principles of the Declaration of Helsinki were followed. All patients underwent genetic counseling and signed a consent form approved by the local ethics committee.

Patients were recruited according to the inclusion criteria as follows: low fertilization (0% < fertilization rate <30%) or TFF (0% fertilization rate) after two cycles and at least four MII oocytes injected per ICSI cycle. Patients with severe male factors (globozoospermia, severe teratozoospermia, and/or <32% progressive motility rate) or abnormal zona pellucida (thick or dark or lacking) in their oocytes were excluded.

### 2.2 Mutation detection and bioinformatics analysis

Mutation detection in the *WEE2*, *TLE6*, and *PLCZ1* genes was performed on the two couples by Sanger sequencing of polymerase chain reaction (PCR) products of all exons and flanking intronic regions using specific primers (the primer sequences and PCR conditions are available on request). The mutations were named according to the Human Genome Variation Society (HGVS) standards (http://www.hgvs.org/mutnomen/) with +1 corresponding to the A of the ATG translation initiation codon in the GenBank cDNA sequence (*WEE2* for NM_001105558.1, *TLE6* for NM_001143986.2, and *PLCZ1* for NM_033123.4). We also sequenced DNA from 200 male control subjects.

**Figure 2:**
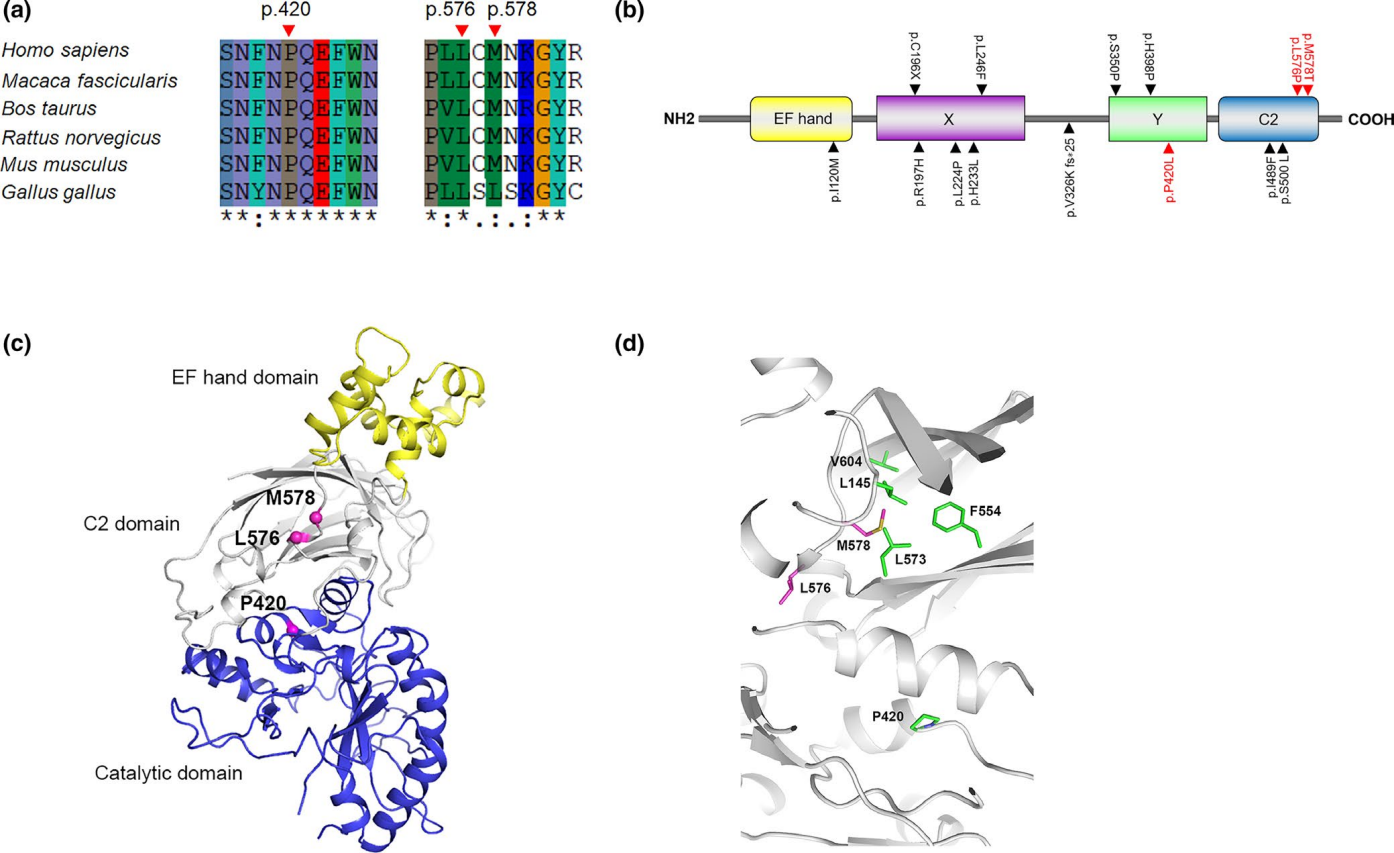
Bioinformatic analysis of novel mutations in *PLCZ1*. (a) Comparison of the human PLCZ1 amino acid sequence with five different species (*Macaca fascicularis*, *Bos taurus*, *Rattus norvegicus*, *Mus musculus*, and *Gallus gallus*). Pro420, Leu576, and Met578 are conserved in PLCZ1. (b) Schematic illustration of the domains in PLCZ1. The wild‐type PLCZ1 protein has 608 amino acids and contains EF hand domain, two catalytic domains (X‐box and Y‐box) and C2 domain. Three novel mutations identified in our study are highlighted in red, and other known mutations found in FF patients are highlighted in black. (c) Overall structure of hPLCZ1. EF hand domain, C2 domain, and catalytic domain are shown as yellow, white, and blue cartoons, respectively. Ca atoms of P420, L576, and M578 are shown as pink spheres. The residue ID is labeled nearby the corresponding residues. (d) Zoom‐in view of hPLCZ1 structure. P420, L576, M578, and hydrophobic core residues are shown as purple and green sticks, respectively. The residue ID is labeled nearby the corresponding residues

CLUSTAL X (1.81) (Thompson, Gibson, Plewniak, Jeanmougin, & Higgins, 1997) was used to compare the human PLCZ1 amino acid sequence (*Homo sapiens* UniProt ID Q86YW0) with those of five other species (*Macaca fascicularis*, *Bos taurus*, *Rattus norvegicus*, *Mus musculus*, and *Gallus gallus*) (Figure 2a). The effects of the sequence variants were predicted using PolyPhen‐2 (http://genetics.bwh.harvard.edu/pph2/), Mutation Taster (http://www.mutationtaster.org/), and SIFT (http://sift.jcvi.org/). The database of the Exome Aggregation Consortium (ExAC) browser (http://exac.broadinstitute.org/), the Combined Annotation Dependent Depletion (CADD) (https://cadd.gs.washington.edu/), and the 1000 Genomes (1000G) browser (https://www.internationalgenome.org/) were used to determine the allele frequencies of the variant.

Information on the motifs and domains of the wild‐type PLCZ1 protein was obtained from UniProt (https://www.uniprot.org) and InterPro (http://www.ebi.ac.uk/interpro/). The 3D structural model of hPLCZ1 was generated by comparative modeling based on the complex structure of rPLCD1, Ca^2+^, and inositol‐1,4,5‐trisphosphate (PDB code: 1DJX) (Essen et al., 1997). The sequence alignment was performed by ClustalX2 (Larkin et al., 2007). hPLCZ1 and rPLCD1 share 45% sequence identity and 65% similarity. Ten models were generated using Modeller v9.14 (Martí‐Renom et al., 2000). The structure with the lowest probability density functions total energy was selected for analysis.

### 2.3 Vector construction, cell culture, and transfection

**Figure 3:**
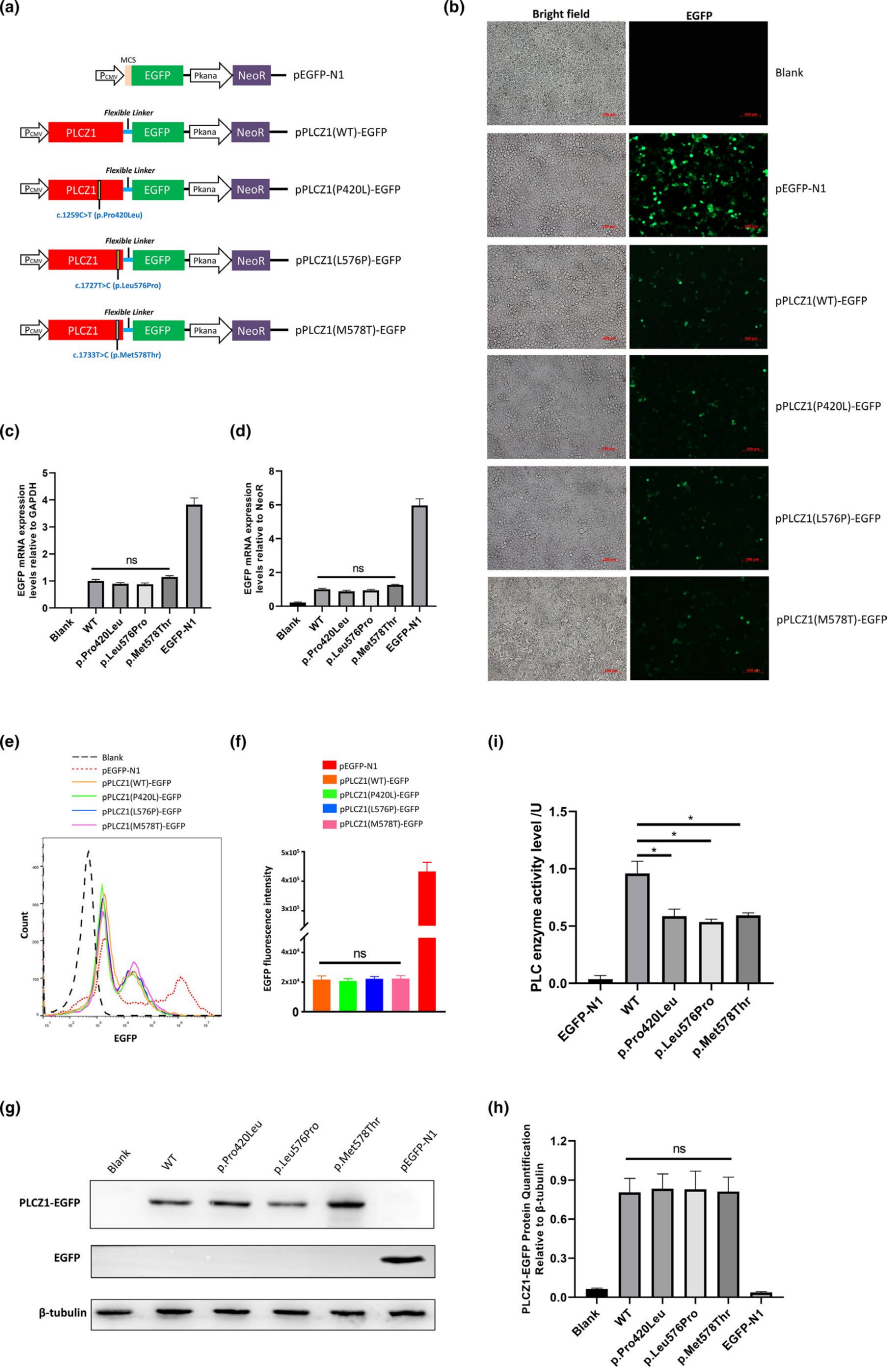
Functional analysis of the identified mutations in PLCZ1. (a) Schematic diagram of plasmids for expressing the wild‐type and mutant PLCZ1 fused with EGFP via a flexible linker. (b) Fluorescence microscopy images of EGFP expression in HEK293T cells 40 hr post transfection. Blank stands for negative control where cells are non‐transfected. Scale bar =100 μm. (c) qRT‐PCR analysis of the transcriptional levels of *PLCZ1* relative to *GAPDH* in transfected HEK293T cells. Blank stands for negative control where cells are non‐transfected. (d) qRT‐PCR analysis of the transcriptional levels of *PLCZ1* relative to *NeoR* in transfected HEK293T cells. Blank stands for negative control where cells are non‐transfected. (e) Representative flow cytometry histogram of EGFP signal of cells 40 hr post transfection. Blank stands for negative control where cells are non‐transfected. (f) Quantification of EGFP fluorescence intensity in transfected HEK293T cells as determined by cytometry analysis. (g) Western blot analysis of EGFP expression levels in transfected HEK293T cells. Blank stands for negative control where cells are non‐transfected. (h) Quantification of PLCZ1‐EGFP fusion protein expression levels based on of the intensity of bands of Western blot. Blank stands for negative control where cells are non‐transfected. (i) Determination of the catalytic activity of over‐expressed the wild‐type and mutant PLCZ1. Asterisks (*) indicate significant differences (*p* < 0.01).

The CDS of wild‐type and mutant human *PLCZ1* followed by a flexible linker (Chen, Zaro, & Shen, 2013) were synthesized by Generay Biotech (China), and then, cloned into the pEGFP‐N1 vector (Clontech, USA) to create the pCMV‐PLCZ1 (WT)‐EGFP, pCMV‐PLCZ1 (P420L)‐EGFP, pCMV‐PLCZ1 (L576P)‐EGFP, and pCMV‐PLCZ1 (M578T)‐EGFP vectors (Figure 3a).

HEK293T cells were cultured in Dulbecco's modified Eagle's Medium (DMEM) with 10% of fetal bovine serum (FBS) (Gibco, USA) without any antibiotics, and then, incubated at 37°C with 5% of CO_2_. The cells were cultured in six‐well plates to reach approximately 1 × 10^6^ cells per well. Then, 3 μg of each plasmid was transfected into the cells using Lipofectamine^®^ 3000 (Invitrogen, USA). The GFP fluorescence signal was detected under a Nikon Eclipse TE2000‐U microscope (Nikon, Japan) 40 hr after transfection.

### 2.4 Flow cytometry

To quantify the GFP fluorescence intensity in the cells, 40 hr after transfection, the HEK293T cells were treated with trypsin and resuspended in PBS. The cells were analyzed with a Beckman CytoFLEX (Beckman Coulter, USA). Data were further analyzed by using FlowJo software (FlowJo v10.0.7).

### 2.5 Quantitative RT‐PCR

Total RNA was prepared from HEK293T cells 40 hr after transfection by using TRIzol reagent (Invitrogen, USA), and then, treated with DNase I (Genstar, China) to avoid DNA contamination. Reverse transcription was performed using StarScript II First‐strand cDNA Synthesis Mix (Genstar, China) with a random primer. The mRNA expression levels of *PLCZ1*, the housekeeping gene *GAPDH* and the reference gene *NeoR* on the expression plasmid were determined by a Roche LightCycler^®^ 480 (Roche, Switzerland). Primers were synthesized by Sangon Biotech (China) (Table S1). The relative mRNA expression level of the target genes was calculated by the 2^−△△C^ *^t^* method.

### 2.6 Western blot analysis

Total protein was prepared from HEK293T cells 40 hr after transfection by using RIPA lysis buffer (Beyotime, China). All protein samples were quantified with a bicinchoninic acid (BCA) assay by using a Pierce™ BCA Protein Assay Kit (Thermo Scientific, USA). Then, 30 µg of each protein sample was separated on a 12% of SDS‐PAGE and transferred onto polyvinylidene fluoride (PVDF) membranes that had been pretreated with methanol. The membranes were blocked with 3% of bovine serum albumin (BSA) in Tris‐buffered saline (TBS) buffer for 2 hr at room temperature. After blocking, the membranes were incubated overnight at 4°C with primary antibodies against β‐tubulin and GFP, respectively. Horseradish peroxidase (HRP)‐conjugated secondary antibodies were used to treat the membranes after incubation with the primary antibodies. After that, an enhanced chemiluminescence (ECL) kit (Fdbio, China) was used for detecting the blotting signals by GelView 6000 Pro (BLT, China). Quantification of the gray value of the bands was conducted by using ImageJ software (ImageJ, v1.50). The target protein expression levels were normalized to the reference protein β‐tubulin.

### 2.7 Determination of the catalytic activity of phospholipase C zeta 1 (PLCZ1)

Catalytic activity was determined by using a phospholipase C (PLC) enzyme activity detection kit (Solarbio, China) following the instructions of the manufacturer. Briefly, total protein containing the overexpressed PLZC1 was prepared from the HEK293T cells 40 hr after transfection of wild‐type and mutant *PLCZ1* expression plasmids using the extraction solution provided by the kit. The substrate natriuretic peptide C (NPPC) was subjected to the extracted protein to be catalyzed into p‐nitrophenol, which can be detected at 410 nm by a microplate reader (BioTek, USA). The enzymatic activity unit was defined as the amount of protein required to hydrolyze natriuretic peptide C (NPPC) to produce 1 nmol of p‐nitrophenol per minute. The enzymatic activity was calculated according to the formula provided by the kit: enzymatic activity (nmol/min/mg protein) = (Absorbance target − Absorbance reference + 0.0103)/0.0095 × *V*_total_/(*V*_sample_ × Concentration)/Time (min).

### 2.8 Statistical analysis

Quantitative data are presented as the mean ± *SEM* of three samples with three parallel repetitions. Differences between means were tested by one‐way analysis of variance (ANOVA) followed by Student's *t* test. (SPSS software, v18.0). The significance level was set at *p* < 0.05.

## 3 RESULTS

### 3.1 Patients and mutation detection

The female patients had essentially normal ovarian reserve abilities, and their husbands also had normal semen parameters. Owing to years of primary infertility, IVF was performed for these patients. Human chorionic gonadotropin (hCG) was administered 36 hr before oocyte pick‐up (OPU) was performed. One family had TFF for two cycles, and another family displayed low fertilization in more than two cycles (Table 1). All oocytes with FF did not form the second polar body (2PB) and 2PN. In the second couple, they acquired advanced fertilization rate and achieved a live birth after ICSI in the third cycle (Table 1).

**Table 1:** Clinical characteristics of affected patients

Coupleno.	Husband age (years)	Semen volume (ml)	Concentration (10^6^/ml)	Motility(PR %)	Morphology (normal %)	Sperm acrosin activity (μIU/M)	Wife age (years)	Cycle (n)	IVF and ICSI attempts	Stimulation protocol	Rretrieved oocytes (n)	MII oocytes (n)	Fertilization oocytes (n)	Normal fertilization oocytes (2PN) (n)	Normal fertilization rate (%)	Reproductive outcome
1	36	2.6	14.6	40.0	4	NA	29	First	IVF	Long	8	6	0	0	0 (0/8)	No ET
	37	3.0	65.0	40.6	4	NA	30	Second	IVF+ICSI	GnRH antagonist	4	4	0	0	0 (0/4)	No ET
2	33	2.3	177.0	35.0	10	NA	35	First	IVF+ICSI	Long	6	6	2	1	16.7 (1/6)	No pregnancy
	33	0.5	50.0	60.0	10	NA	35	Second	IVF+ICSI	GnRH antagonist	7	6	2	2	28.6 (2/7)	No pregnancy
	34	1.5	130.0	60.0	10	NA	36	Third	ICSI	Ultra‐long	14	13	6	6	46.2 (6/13)	Live birth

Note

Semen volume (lower reference limit: 1.5 ml), The total sperm concentration (lower reference limit: 15 × 10^6^/ml), Progressive motility rate (PR) (lower reference limit: 32%), Normal sperm morphology (lower reference limit: 4%), Sperm acrosin activity (normal range 48.20–218.70 μIU/M) (World Health Organization, 2010).

Abbreviations: ET, embryo transferred; ICSI, intracytoplasmic sperm injection; MII, metaphase II; NA, data not available or without experimental data.

Direct sequencing of the *WEE2* and *TLE6* genes initially revealed no mutations in the wives of the two couples. We then examined the *PLCZ1* gene in the husbands. Sanger sequencing of the *PLCZ1* gene in the husband (II:3) of the couple 1 (Figure 1a) displayed novel compound heterozygous mutations of c.1259C>T (p.P420L) and c.1733T>C (p.M578 T) (Figure 1c,e). Sequencing of his parental DNA showed that his father was the carrier of c.1733T>C (p.M578T) and his mother of c.1259C>T (p.P420L). In the second couple (Figure 1b), a novel missense mutation, c.1727T>C (p.L576P) was found in *PLCZ1* gene in husband (II:1) (Figure 1d), but genetic information of other family members is not available. Furthermore, we examined 200 healthy male control subjects (400 alleles) and searched ExAC and 1000G and CADD databases, and we found that these three novel variants in *PLCZ1* were absent from our cohort and the database, suggesting that it might not be a benign polymorphism (Table 2).

**Table 2:** *PLCZ1* gene mutations detected in patients

Mutations (het/homo)	Exon	Mutation type	ExAC^a^	1000G^a^	CADD^a^	Our control cohorts^a^	PolyPhen‐2^b^	SIFT^b^	Mutation taster^b^
c.1259C>T (p.P420L) het	11	Missense	NA	NA	NA	0 (0/400)	Probably damaging	Damaging	Disease causing
c.1727T>C (p.L576P) homo	14	Missense	NA	NA	NA	0 (0/400)	Probably damaging	Tolerated	Disease causing
c.1733T>C (p.M578T) het	14	Missense	NA	NA	NA	0 (0/400)	Possibly damaging	Damaging	Disease causing

Abbreviations: het, heterozygote; homo, homozygote; NA, data not available.

^a^ Allele frequency in ExAC (the Exome Aggregation Consortium), 1000G (the 1000 Genomes), CADD (the Combined Annotation Dependent Depletion), and our control cohorts.

^b^ Mutation assessment by PolyPhen‐2, SIFT, Mutation Taster.

### 3.2 Bioinformatics analysis

The computational programs SIFT, PolyPhen‐2, and Mutation Taster predicted that the effects of these variants were deleterious (Table 2). Alignment of the PLCZ1 protein showed the proline, leucine, and methionine at the 420st, 576st, and 578st positions, respectively, were highly conserved among the six species (Figure 2a). All of the mutations occurred in the Y catalytic and C2 domains (Figure 2b).

Moreover, we constructed a 3D structural model for human PLCZ1 based on the crystallographic structure of rat PLCD1 (Essen et al., 1997). As shown in Figure 2c, hPLCZ1 is composed of a tandem EF hand domain, a catalytic domain, and a C2 domain. The C2 domain is responsible for Ca^2+^ binding and interactions with activated Gαq, and its structural stability may play a critical role in hPLCZ1 activity (Essen et al., 1997; Waldo et al., 2010). L576 was located in a short α‐helix of the C2 domain, which was at the interface between C2 and the catalytic domain. The helix propensity of proline is the lowest among all residues (Pace & Scholtz, 1998). As a helix disruptor, proline at this site might prevent helix formation (Figure 2d). Therefore, the L576P mutation might affect both the C2 domain structure and the C2‐catalytic domain interaction.

In contrast, P420 was located at the end of a short helix in the catalytic domain. The P420L mutation might enhance the local helix propensity and interfere with structural stability. The side chain of M578 was deeply buried in the hydrophobic core of the C2 domain (Figure 2d). The hydrophobic interactions between M578 and L145, F554, and L573 as well as V604 are important to maintain the stability of the C2 domain. The mutation of M578 to a hydrophilic threonine could severely destabilize the hydrophobic core, leading to PLCZ1 dysfunction.

### 3.3 In vitro functional analysis

In this study, we coexpressed the wild‐type or mutant CDS of PLCZ1 in an EGFP reporter using a flexible linker (Figure 3a). Thus, the expression level of EGFP should be equivalent to PLCZ1, and the expression level of wild‐type or mutant PLCZ1 was easily detected by measuring the EGFP signal intensity. We detected the fluorescence signal of EGFP in HEK293T cells under a fluorescence microscope 40 hr after transfection. We found that the EGFP fluorescence signal in the transfected cells had no differences between wild‐type PLCZ1 expression vector pCMV‐PLCZ1 (WT)‐EGFP and mutant PLCZ1 expression vectors pCMV‐PLCZ1 (P420L)‐EGFP, pCMV‐PLCZ1 (L576P)‐EGFP, and pCMV‐PLCZ1 (M578T)‐EGFP (Figure 3b).

To confirm that the fluorescence results were reliable, we used both the GAPDH housekeeping gene and the NeoR gene driven by an independent promoter on the plasmid of the PLCZ1 expression vector (Figure 3a) as reference genes to determine the transcription level of wild‐type and mutant PLCZ1. The results showed that all of the three missense mutations (P420L, L576P, and M578T) had no impact on the transcriptional expression of PLCZ1 (Figure 3c,d). Flow cytometry analysis confirmed that the expression of EGFP was not different between the wild‐type group and the mutant groups (Figure 3e,f). The Western blot analysis of EGFP also revealed that there were no differences between the wild‐type group and mutant groups (Figure 3g,h). However, the enzymatic activity analysis indicated that all three missense mutations significantly impaired the catalytic activity of PLCZ1 (*p* < 0.01) (Figure 3i).

## 4 DISCUSSION

The clinical incidence of TFF in IVF is extremely low, and it is even lower for ICSI (Lee, Lee, Park, Yang, & Lim, 2017; Tosti & Menezo, 2016). There is no guideline providing a definition of the cutoff values for FF. Some IVF centers identify low fertilization as 20% fertilization (Dai et al., 2019), and others as 25% (Dai et al., 2020; Shinar et al., 2014). Our center considers low fertilization a fertilization rate under 30%, and TFF is a 0% fertilization rate per cycle according to our in‐house data. In this study, we characterized two families with similar clinical phenotypes: low fertilization or TFF observed in oocytes without 2PB extrusion and 2PN formation during repeated IVF and ICSI cycles after excluding phenotypic abnormalities of the sperms and oocytes. It was believed that AOA was a suitable technique for couples with FF (a fertilization rate below 30%) (Yeste et al., 2016), and in particular, that it could succeed in FF caused by PLCζ errors (Dai et al., 2020; Torra‐Massana et al., 2019). In our study, the first couple did not select AOA treatment in the third ICSI cycle, although we had told them the pros and cons.

Generally, the major mechanism of fertilization failure is attributed to oocyte activation deficiency, which is classified into sperm‐ and oocyte‐related OAD (Yeste et al., 2016). In addition to abnormal morphology of the sperm or a thick zona pellucida of oocytes probably causing FF and 2PN arrest, known genetic factors including sperm‐related defects of the *PLCZ1* gene and oocyte‐related defects of the *TLE6* and *WEE2* genes have been reported to underlie this form of the disease (Alazami et al., 2015; Escoffier et al., 2016; Sang et al., 2018). PLCζ, as a key sperm‐borne oocyte activation factor (SOAF), can elicit Ca^2+^ oscillations within the oocyte by hydrolyzing phosphatidylinositol 4,5‐bisphosphate (PIP2) from plasma membrane sources into inositol 1,4,5‐trisphosphate (InsP3) and diacylglycerol (DAG) (Torra‐Massana et al., 2019; Yeste et al., 2016). In our study, the husband in couple 2 carrying the homozygous mutations (p.L576P) in PLCZ1 that resulted in low fertilization had a live birth without AOA treatment in the third cycle, but the first couple with compound heterozygous mutations (M578T and P420L) showed TFF.

In the structure of the mutant proteins in Figure 2c–d, the L576P mutation was located at the interface between C2 and the catalytic domain, and it might cause milder damage in the C2 domain or C2‐catalytic domain interaction than M578T, which was deeply buried in the hydrophobic core of the C2 domain and could severely destabilize the hydrophobic core, leading to PLCZ1 dysfunction. The other novel mutation (p.P420L) in our study was located in the Y catalytic domain, which was a highly conserved region of PLCζ and was absolutely essential for PIP2 hydrolysis (Suh et al., 2008). Therefore, we supposed that the L576P mutation might result in the partial loss of function of PLCZ1, but M578T combined with P420L might lead to complete loss of function.

Moreover, as PLCZ1 is mainly expressed in the testis, it plays an important role during fertilization that leads to early embryonic development. A functional PLCZ1 with its own promoter generally will not be expressed in HEK293T. Therefore, in this study, we expressed PLCZ1 CDS under the control of the strong promoter CMV in HEK293T cells to analyze the effect of the mutation on the expression of PLCZ1. Our results revealed that the investigated three missense mutations did not affect the transcription and translation of PLCZ1, which is consistent with that observed in other proteins (Lamborn et al., 2017). However, further functional detection revealed that all of the mutations, which occurred in the Y catalytic and C2 domains (Figure 2b–d), significantly impaired the catalytic activity of PLCZ1 (Figure 3i). We hypothesize that these mutations would lead to an inefficient elicitation of Ca^2+^ oscillations and egg activation during fertilization, eventually leading to fertilization failure (FF). According to the 2015 ACMG Standards and Guidelines (Richards et al., 2015), the three novel mutations could be classified as pathogenic.

In conclusion, we successfully identified three novel mutations in *PLCZ1* as causes of low fertilization and TFF in two couples with an autosomal recessive inheritance pattern. Our findings further expand the spectrum of *PLCZ1* mutations and provide new evidence for the genetic basis of FF.

## 5 ETHICS APPROVAL AND CONSENT TO PARTICIPATE

The study protocol and all subjects who participated in this study were approved by the Institutional Review Board of our institute, and informed consent was obtained from all patients prior to their participation in accordance with institutional and national guidelines.

## 6 COMPETING INTEREST

The authors declare that they have no competing interests.

## AUTHOR CONTRIBUTIONS

WJW and PY were responsible for the study design and final editing of the paper. PY performed the genetic study and partial in silico analyses. PY and LYZ provided the patients’ blood collected and analyzed clinical data. HL and LHL performed the bioinformatic analysis. ZYH and QYL performed the in vitro functional analysis. SBO and YQZ provided embryo observation and research. PY and HL and ZYH drafted the manuscript. QXZ and WJW revised the manuscript. All authors read and approved the final manuscript.

## Supporting information

Table S1

## Data Availability

The data that support the findings of this study are available from the corresponding author upon reasonable request.
