# Supplementary material for: Novel mutations in the PLCZ1 gene associated with human low or failed fertilization
Source: Mol Genet Genomic Med. 2020 Aug 24;8(10):e1470. doi: 10.1002/mgg3.1470 (PMC7549595; doi:10.1002/mgg3.1470)
Supplement: Supplementary file 1 — Table S1 [file MGG3-8-e1470-s001.docx]

**Supplement Table 1. Primers used for qRT-PCR analysis.**

| **Primer name** | **Sequence (5’to 3’)** |
| --- | --- |
| EGFP F | CGACGTAAACGGCCACAAGT |
| EGFP R | TGCTTCATGTGGTCGGGGTA |
| NeoR F | TTGAACAAGATGGATTGCACGC |
| NeoR R  GAPDH F  GAPDH R | GCCTCGTCTTGCAGTTCATTCA  GTCGGAGTGAACGGATTT  GATGACAAGCTTCCCGTTC |
